# Data Inaccessibility Is Stifling the Digital Twin Implementation in Health Care

**DOI:** 10.2196/76524

**Published:** 2025-06-03

**Authors:** Md Doulotuzzaman Xames

**Affiliations:** 1 Grado Department of Industrial and Systems Engineering, Virginia Tech, 1145 Perry St, Blacksburg, Virginia, 24060, United States, 1 5405585848

**Keywords:** digital twins, health IT, implementation challenges, data accessibility, data governance

 The recent meta-review by Ringeval et al [1] offers a useful synthesis of digital twin (DT) applications in health care, highlighting DTs’ emerging value in personalized medicine, operational efficiency, and medical research. Their categorization of implementation challenges, including data quality, ethical governance, and socioeconomic disparities, represents a meaningful step toward addressing barriers to adoption. However, what remains underemphasized is a foundational obstacle to translational DT research: the systemic inaccessibility of high-fidelity clinical and operational data.

As a researcher developing a DT to monitor and manage health care provider workload—part of a case study at a US primary care facility—our team has repeatedly encountered delays and disruptions due to institutional review board bottlenecks, fragmented governance systems, and restrictive data ownership policies. These challenges are not just bureaucratic inconveniences; they introduce epistemic uncertainty into model development, undermine calibration and validation efforts, and threaten the scalability of DT systems in clinical settings. Notably, Ringeval et al [1] recognize data-related challenges, but their analysis remains largely conceptual to reflect the practical difficulties faced by DT implementation teams.

Health care DTs inherently depend on granular, individualized, and real-time data flows to simulate complex physiological or behavioral systems. As emphasized by Corral-Acero et al [2], real-time synchronization between physical and digital entities is a defining feature of the DT paradigm. Yet, such synchronization cannot occur without reliable and timely access to data—an issue too often neglected in theoretical discussions. Even “virtual patient” constructs—discussed elsewhere as privacy-preserving alternatives—require baseline access to real-world patient data, which remain sequestered within institutional silos.

The scale of this access problem has been recognized in national-level assessments. The National Academies underscore that the integration of data from heterogeneous sources in DT systems is impeded by strict data access and a lack of collaboration [3]. These barriers are exacerbated by regulatory, frameworks that, in many cases, have not evolved to support the dynamic, high-frequency data requirements of modern machine learning and complex systems modeling approaches. As Terranova and Venkatakrishnan [4] note, model-informed precision medicine relies on timely, granular data to capture disease trajectories and treatment responses; delays in accessing such data not only hinder innovation but also inject risk into clinical decision-making.

To be clear, technical solutions exist. Federated learning, differential privacy, and blockchain-enabled data governance can support secure, distributed modeling while respecting patient privacy [5]. However, these innovations have struggled to gain traction not due to technical immaturity but because of institutional inertia, legal ambiguity, and a lack of incentives for change. The issue is no longer whether we can share data securely but whether health care institutions are willing and are enabled to do so.

If the transformative potential of DTs described by Ringeval et al [1] is to be realized, bold reforms in data governance must be prioritized. From an implementation science perspective, access to high-resolution, real-time data is not a peripheral technical detail; it is a scientific and ethical imperative. Without addressing this bottleneck, DTs will remain more aspirational than actionable.

## Abbreviations

DT: digital twin
